# IRGM1 enhances B16 melanoma cell metastasis through PI3K-Rac1 mediated epithelial mesenchymal transition

**DOI:** 10.1038/srep12357

**Published:** 2015-07-23

**Authors:** Linlu Tian, Lixian Li, Wenjing Xing, Rui Li, Chunying Pei, Xiao Dong, Yanran Fu, Changcong Gu, Xize Guo, Yulong Jia, Guangyou Wang, Jinghua Wang, Bo Li, Huan Ren, Hongwei Xu

**Affiliations:** 1 Department of Immunology, Heilongjiang Provincial Key Laboratory for Infection and Immunity, Harbin Medical University, Harbin 150081, China; 2 Department of Neurosurgery, The Affiliated First Hospital of Harbin Medical University, Harbin 150001, China; 3 Neuroimmunology Unit, Montreal Neurological Institute, McGill University, Montreal H3A2B4, Canada; 4 Department of Neurobiology, Harbin Medical University, Harbin 150081, China

## Abstract

Melanoma is one of the most aggressive skin cancers and is well known for its high metastatic rate. Studies have shown that epithelial mesenchymal transition (EMT) is essential for melanoma cell metastasis. However, the molecular mechanisms underlying EMT are still not fully understood. We have shown that IRGM1, a member of immunity-related GTPase family that regulates immune cell motility, is highly expressed by melanoma cells. The current study aimed to explore whether and how IRGM1 may regulate melanoma cell metastasis. To test this, we modified IRGM1 expression in B16 melanoma cells. We found that over-expression of IRGM1 substantially enhanced pulmonary metastasis *in vivo*. In keeping with that, knocking-in IRGM1 strongly enhanced while knocking-down IRGM1 impaired B16 cell migration and invasion ability *in vitro*. Interestingly, we observed that IRGM1 enhanced F-actin polymerization and triggers epithelial mesenchymal transition (EMT) through a mechanism involved in PIK3CA mediated Rac1 activation. Together, these data reveals a novel molecular mechanism that involved in melanoma metastasis.

Melanoma is the most aggressive skin cancer and well known for its poor prognosis and low survival rate^1^. The high rate of metastasis is the main cause of death in the patients with melanoma^1^. Epithelial mesenchymal transition (EMT) is a rate limiting step during melanoma metastasis^2^,^3^. However the molecular mechanisms of EMT is still not fully understood.

IRGM1 belongs to Immune-related GTPase family^4^. The role of IRGM1 in regulating autophagy-mediated cellular survival has been extensively studied in many cell types^5^,^6^,^7^,^8^, including our recent report in melanoma cells during nutrient deprivation^9^. Recent data suggested that IRGM1 was involved in immune cell motility during condition that autophagy was not generally triggered^10^,^11^, highlighting an autophagy independent role of IRGM1. However, the mechanisms of IRGM1 in regulating cell migration are not clear.

In the current study, we studied the role of IRGM1 in melanoma metastasis. We showed that IRGM1 substantially increased B16 melanoma cells metastasis both *in vivo* and *in vitro*. We further demonstrated that IRGM1 enhanced Rac-1 mediated F-actin polymerization and induced epithelial mesenchymal transition (EMT) in B16 cells. Knocking down PIK3CA strongly diminished IRGM1 mediated Rac1 activation and cell migration and invasion. Together, these data revealed a novel function of IRGM1 in melanoma metastasis and provides a potential therapeutic target to limit melanoma metastasis.

## Result

### IRGM1 enhances melanoma pulmonary metastasis *in vivo*

**Figure 1:**
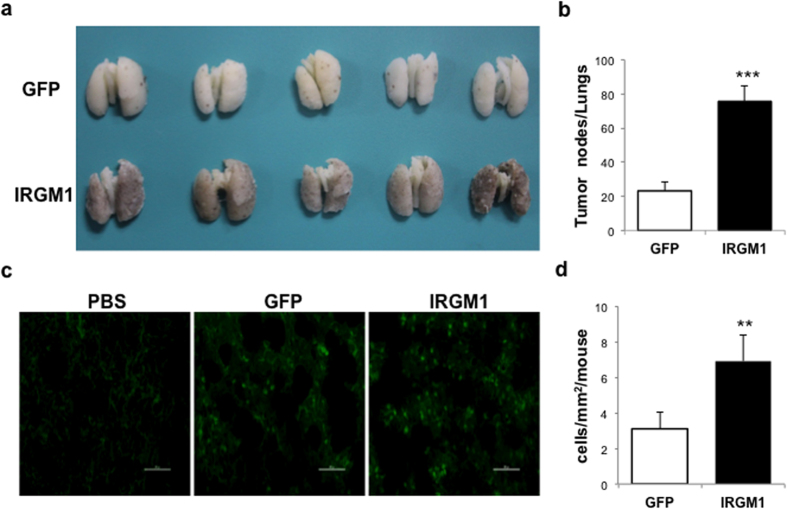
IRGM1 enhanced melanoma pulmonary metastasis *in vivo*. B16 cells were transduced with either GFP or IRGM1-GFP lentiviral vectors. (**a**,**b**) Cells (2 × 10^5^ cells) were intravenously inoculated into C57BL/6 mice. The mice were scarified at day 15 to evaluate the ability of metastasis by counting the number of tumor nodules in the lungs. (**c**,**d**) GFP or IRGM1-GFP B16 melanoma cells were injected into left cardiac ventricle. Mice were scarified at day 5. GFP^+^ B16 cells in the lung were counted by Image J. (Unpaired t-test, n = 7/group, **p < 0.01, ***p < 0.001).

To explore the potential role of IRGM1 in melanoma metastasis *in vivo*, we first injected C57/B6 mice with either GFP or IRGM1-GFP lentiviral vector transduced B16 melanoma cells intravenously to induce the mice model of melanoma pulmonary metastasis. We observed that compared with GFP group, over-expression of IRGM1 substantially increased the number of tumor nodules in lungs (Fig. 1a,b) at day 15. To further confirm this observation, GFP tagged IRGM1 transduced or control B16 cells were directly injected into left cardiac ventricle. Consistently, we observed an increased number of GFP^+^ B16 cells in the lungs of IRGM1-GFP group as compared to control GFP group at day 5 (Fig. 1c,d). Previously, we have reported that IRGM1 mediated autophagy is important for the survival of melanoma cells during nutrient deprivation^9^. Autophagy activity was barely detectable at stage when we sacrificed the mice (data not shown), suggesting that the impact of IRGM1 on melanoma metastasis maybe autophagy independent.

### IRGM1 promoted B16 cell migration and invasion

**Figure 2:**
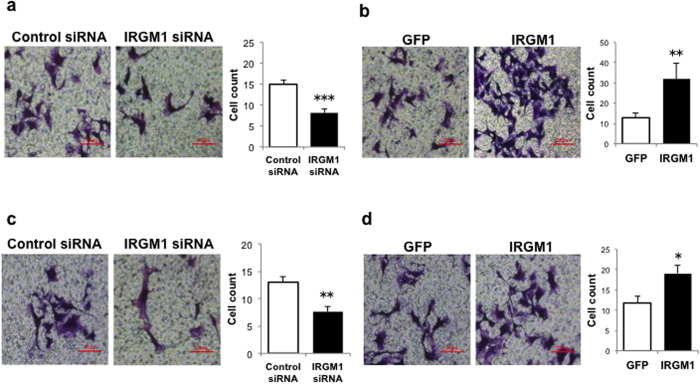
IRGM1 promotes B16 melanoma cell migration and invasion. B16 cells were transfected with either control or IRGM1siRNA (**a**,**c**) or transduced with either GFP or IRGM1-GFP lentiviral vectors (**b**,**d**). (**a**,**b**) B16 cells (5 × 10^3^) were then seeded on top of the Boyden chambers. After 24 hrs, B16 cells on the bottom were stained with 1% crystal violet. (**c**,**d**) B16 cells (5 × 10^3^) were seeded into Boyden chambers coated with Matrigel. After 48 hrs, cells on the underside of the membranes were fixed and then stained with crystal violet. Y-axis represents stained cell count per field. Data represent three independent experiments. (Unpaired t-test; *p < 0.05, **p < 0.01, ***p < 0.001).

Next, we performed boyden chamber based migration & invasion assay to validate the *vivo* observation above. IRGM1 expression was modified by specific siRNA (knocked-down) or IRGM1-GFP lentivirus (knocked-in). Knocking down IRGM1 remarkably reduced B16 cells migration (Fig. 2a) while overexpressing IRGM1 in B16 cells showed enhanced capacity to migrate as compared to control B16 cells (Fig. 2b). In order to test the impact of IRGM1 on invasion, Matrigel was pre-coated on the membrane of the boyden chamber. Consistent with the migration assay, we found that knocking down IRGM1 strongly decreased (Fig. 2c) while overexpressing IRGM1 increased the number of cells that passed through the chamber (Fig. 2d). These data indicates that IRGM1 is an important mediator for B16 cells migration and invasion.

### IRGM1 promotes epithelial mesenchymal transition (EMT)

**Figure 3:**
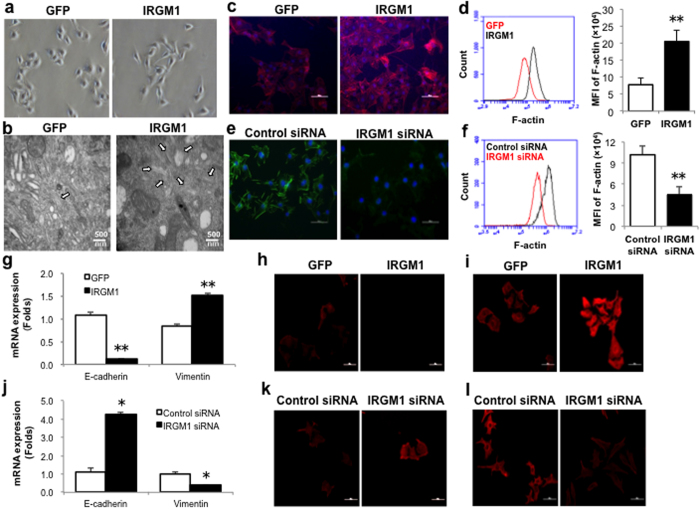
IRGM1 enhances epithelial mesenchymal transition (EMT) of B16 cells. B16 cells were transduced with IRGM1-GFP lentiviral vectors (**a**-**d**, **g**-**i**) or tranfected with IRGM1 siRNA to modulate IRGM1 expression (**e**-**f**, **j**-**l**). (**a**). EMT morphological change of B16 cells induced by over-expression of IRGM1 were observed by the optical microscope. (**b**). Microfilaments structure in either control GFP or IRGM1-GFP B16 Cells were detected by transmission electronic microscope (TEM). Phalloidin was used to detect F-actin expression in IRGM1 over-expressing or knocking-down B16 cells and the fluorescence signals were captured by the confocal microscope (**c**, **e**) and quantified by flow cytometry (**d**, **f**). The expression of E-cadherin and Vimentin was detected by Real-time PCR (**g**, **j**) and immunofluroscence staining (**h**-**I**, **k**-**l**). Data represent at least three independent experiments. (student t test, *p<0.05, **p<0.01)

The results above suggested a role of IRGM1 in regulating B16 cell metastasis. Studies have shown that EMT is important for cancer cell metastasis^12^,^13^. Typical characteristics in tumor cells during EMT include the change of cell morphology, cytoskeleton rearrangement such as F-actin polymerization and reciprocal change of several adhesion molecules such as E-cadherin and Vimentin^14^,^15^. We first assessed cell morphology. Notably, in contrast to parental cells, which had a tightly packed epithelial-like morphology, overexpression of IRGM1 in B16 cells results in a fibroblastic spindle-shape morphology reminiscent of the epithelial mesenchymal transition (EMT) (Fig. 3a). Previous data from our lab have shown that IRGM1 promotes F-actin polymerization^11^. To test whether IRGM1 may also involved in regulating B16 cell F-actin polymerization, we monitored cytoskeleton structure by using transmission Electronic microscope. In keeping with our hypothesis, IRGM1 over-expressing B16 cells showed remarkable increased microfilament structure in the rim of cytoplasm (Fig. 3b). Meanwhile, phalloidin staining revealed that overexpression of IRGM1 strongly increased F-actin synthesis in B16 cells (Fig. 3c,d). In contrast, knocking down IRGM1 significantly decreased the expression levels of F-actin (Fig. 3e,f).

The reciprocal regulation of E-cadherin and Vimentin is another important markers for cells undergoing EMT^14^. Next, we detected the expression of E-cadherin and Vimentin by real time PCR and immunofluorescence assay in IRGM1 overexpressing B16 cells. We found that over-expression of IRGM1 significantly reduced E-cadherin but enhanced Vimentin expression (Fig. 3g–i). The opposite results were observed in the IRGM1 knocked-down B16 cells (Fig. 3j–l). We conclude that IRGM1 induces EMT of B16 cells.

### IRGM1 activates Rac1 through PIK3CA

**Figure 4:**
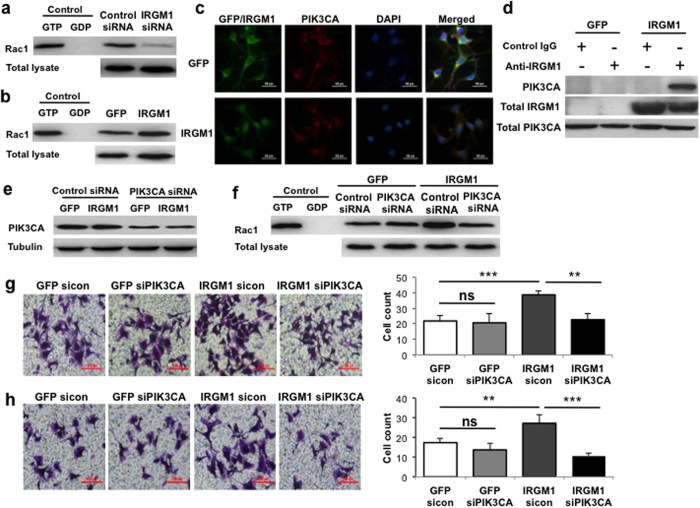
IRGM1 activates PI3K-Rac1 signaling pathway. B16 cells were transfected with either control or IRGM1siRNA (**a**) or transduced with either GFP or IRGM1-GFP lentiviral vectors (**b**–**h**). (**a**,**b**) Rac1 activity was measured in total lysates by GST-PAK-PBD pull down and Rac1 levels were measured by western blot. (**c**) Co-localization of PIK3CA with IRGM1 was determined by immunofluorescence staining in IRGM1 overexpressing B16 cells (Upper panel: GFP; lower panel: IRGM1-GFP; green: IRGM1-GFP/GFP; red: PI3KCA; blue: nuclei). (**d**) Co-IP study was performed to further confirm the co-localization of IRGM1 and PIK3CA. Anti-IRGM1 polyclonal antibody or non-immune IgG (control) were used to pull down IRGM1 from total cell lysates. Anti-PIK3CA monoclonal antibody was used to detect participated PIK3CA. GFP and IRGM1 overexpressing B16 cells were transfected with either control or PIK3CA siRNA (**e**–**h**). (**e**) PIK3CA levels were measured by western blot. (**f**) Rac1 activity was measured in total lysates by GST-PAK-PBD pull down and Rac1 levels were measured by western blot. The migration (**g**) and invasion (**h**) ability of GFP and IRGM1 overexpressing B16 cells were evaluated by using boyden chamber. Data represent three independent experiments. (Unpaired t-test; **p < 0.01, ***p < 0.001)

The results above demonstrate that IRGM1-mediated-EMT promotes melanoma cell metastasis. We tempted to explore the potential mechanism. Rac1, a member of Rho GTPase family, is important for cytoskeleton rearrangement and other molecular events in EMT^16^,^17^. Using active GST-PAK pull down assay, we detected Rac1 activation in either IRGM1 knocked down or knocked in B16 cells. IRGM1 strongly induced Rac1 activation without influence the expression of Rac1 (Fig. 4a,b), suggesting that IRGM1 may regulate F-actin polymerization and EMT through activating Rac1.

Previous data suggested that IRGM1 is one of the regulators for PI3K^18^. PI3K has been reported can activate Rac1^19^,^20^,^21^. We speculated asked whether IRGM1 regulates the activation of Rac1 via PI3K. Using confocal microscopy, we observed that IRGM1 was co-localized with PIK3CA, a catalytic subunit of PI3K, in the IRGM1 over-expressing B16 cells (Fig. 4c). Co-immunoprecipitation assay further confirmed a physical connection between IRGM1 and PIK3CA in B16 cells (Fig. 4d). More importantly, compared with control GFP B16 cells, knocking down PIK3CA in IRGM1 over-expression cells (Fig. 4e) significantly impaired IRGM1 mediated Rac1 activation (Fig. 4f) and cells migration & invasion (Fig. 4g–h). These data together indicates that IRGM1 regulates melanoma metastasis through PI3K-Rac1 pathway.

## Discussion

Here, we have demonstrated that IRGM1 promotes melanoma metastasis. *In vitro*, IRGM1 enhances B16 cell migration and invasion via the induction of F-actin polymerization and EMT. Furthermore, we showed that IRGM1 interacts with PIK3CA, which in turn activates Rac1 to rearrange cytoskeleton and induce EMT. Our study revealed a novel molecular mechanism that contribute to melanoma metastasis and expanded our knowledge regarding the multiple roles of IRGM1 in melanoma.

Metastasis is the leading cause of mortality in melanoma^22^. EMT is a vital step during the early stage of metastasis. The transition from epithelial to mesenchymal allows cancer cells to invade basal membrane entering circulation system and facilitate tumor cell metastasis^15^. Increasing evidence suggested that PI3K signaling pathway is required for melanoma metastasis and involved in EMT in melanoma cells^23^,^24^. PIK3CA, the catalytic subunit of PI3K, is involved in Rac1 activation^25^,^26^. Constant Rac1 activation has been observed in multiple tumor cell lines including melanoma cell^27^. Our previous data has shown that IRGM1 is highly expressed by human melanoma cells *in situ*^9^, suggesting IRGM1 may be one of the contributors that maintain Rac1 activation in melanoma cells.

IRGM1 is well known for its role in regulating autophagy^28^. IRGM1 mediated autophagy has been implicated in many diseased conditions including host defense against bacteria^29^,^30^, virus^31^ and parasite^32^,^33^, autoimmunity^6^,^34^,^35^,^36^ and ischemia^8^. Recently, we reported that through bif-1, IRGM1 induces melanoma cell autophagy and hence enhancing cell survival during nutrient deprivation^9^. Emerging evidence from our lab and others also suggested an autophagy independent role of IRGM1 in cytoskeleton rearrangement in the condition where autophagy was not triggered^10^,^11^. In the current study, we confirmed such observation in B16 melanoma cells, which suggested context dependent functions of IRGM1 in promoting tumorigenesis.

In conclusion, these evidence together with our recent report suggested that autophagy dependent and independent role of IRGM1 are involved in melanoma survival and metastasis, which makes IRGM1 a potential valuable therapeutic target that can limit melanoma growth as well as metastasis.

## Materials and Methods

### Animals and model

C57BL/6 (B6) mice were purchased from HFK Bioscience. All mice were housed in the animal facilities of the Harbin Medical University. All experimental procedures involved were performed according to protocols approved by the Institutional Animal Care and use Committee at the Institute of Genetics and Developmental Biology. Mice model of melanoma was induced as described before^28^. Briefly, C57/B6 mice were injected intravenously with 2 × 10^5^ GFP or GFP tagged IRGM1 B16 cells in 200ul PBS. To quantify tumor nodules, mice were sacrificed at day 15. Lungs were bleached by Fekete’s solution to easily visualized tumor nodules. For the short-term tumor metastasis model, GFP or GFP tagged IRGM1 B16 cells were injected into left cardiac ventricle and sacrificed at day 5. GFP^+^ cells were detected by fluorescence microscope and counted by image J (NIH).

### Cell culture

B16 cells were purchased from ATCC. Cells were cultured in RPMI 1640 medium supplement with 10% FBS at 37 °C in 5% CO2 incubator.

### siRNA and lentivirus transfection/transduction

IRGM1, PIK3CA and negative control siRNA duplexes were synthesized and purified by Invitrogen. The sequence of IRGM1 were as follows: RNAi-1 5′-GGUUACCU GAGGUCAGUAGtt-3′, 5′-CUACUGACCUCAGGUAACCtg-3′; RNAi-2 5′-GGAA AACUACUGGAACUGGtt-3′, 5′-CCAGUUCCAGUAGUUU UCCtt-3′; and RNAi-3 5′-GACCCUUUAUGGCACUUAUtt-3′, 5′-AUAAGUGCCAUAAAGGGUCtt-3′. The sequence of PIK3CA were as follows: RNAi-1 5′-GGGACCCACUAUCUGA AAUTT-3′, 5′-AUUUCAGAUAGUGGGUCCCTT-3′; RNAi-2 5′-GCGUAACUAU UCCUGAAAUTT-3′, 5′-AUUUCAGGAAUAGUUACGCTT-3′; and RNAi-3 5′-GG AGAGACAUCUACGAAAUTT-3′, 5′-AUUUCGUAGAUGUCUCUCCTT-3′. To achieve transient suppression of IRGM1 and PIK3CA expression, the duplex siRNA were transfected into B16 cells. Briefly, 2.5 × 10^4^/well B16 cells were seeded into 24-well plates in complete 1640 medium overnight. Then the cells were transfected with 80 nM siRNA using the N-TER Nanoparticle siRNA transfection reagent (Sigma, N2913), according to the manufacturer’s instructions. After 48 h of siRNA transfection, the protein was extracted with RIPA buffer for detecting the efficacy of down-regulation through western blotting. IRGM1 gene was constructed into lentiviral vectors GV218 by Genechem (Shanghai, China). For lentivirus infection and establishment of stable cell clones, IRGM1-lentivirus and green fluorescent protein (GFP)- lentivirus were transfected into the B16 cells with a multiplicity of infection ranging from 30 to 50 in the presence of polybrene (5 μg/ml). Puromycin (1 μg/ml) was used to select IRGM1-overexpressing B16 cells.

### *In vitro* migration and invasion assay

4-well cell culture inserts which contained 8 um pore size polyethylene terephthalate membrane (Costar) were used to measure cell migration and invasion as described before^29^,^30^. For the invasion assay, the membrane of the cell culture insert was covered with a film of Matrigel (BD). At 48 hrs, cells on the underside of the membranes were fixed in methanol and stained with crystal violet.

### Immunoflurescence staining

The sections were blocked for 1 hour with 10% normal goat serum, 0.3% BSA, 0.05% saponin and 0.3% Triton X-100 in PBS. Then the primary antibodies were added and incubated at 4 °C overnight. The sections were later washed with PBS and incubated with fluorescence-labeled secondary antibody. The sections were then rinsed in PBS and mounted with GEL/Mounting Medium (MO1, Biomeda, USA). Fluorescence microscope (Nikon, Japan) and confocal microscope (Zeiss, Germany) were used to capture the images.

### Phalloidin staining

Cells were fixed with cold 4% paraffin in PBS for 10 min on ice and then perforated with 0.1% trion-X100 in PBS for 30 min. After one time wash with cold PBS, cells were then stained for F-actin with phalloidin– FITC (sigma) or -Texas red (Thermo) for 30 min in 4 °C. After two times washes with PBS, cells were analyzed by Accuri6 cytometry (BD).

### Immunoblotting and Co-immunoprecipitation

Western blotting was used to measure the expression of IRGM1, PIK3CA, Rac1, and tubulin in B16 cell. Antibodies used for Western blot include anti-IRGM1 pAb (AbMart), anti-PIK3CA pAb (proteintech), anti-Rac1 pAb (Cell Signaling), anti-tubulin (Sigma), anti-mouse IgG and anti-rabbit IgG (Cell Signaling). For Co-IP, total cell lysates was incubated with anti-IRGM1 polyclonal antibody (AbMart) at 4 °C overnight. Then the antibody-antigen complex was captured by Protein A/G agarose beads (Santa Cruz Biotechnology). PIK3CA pAb was used to measure the binding signal. Blots were developed with Super Signal West Femto Maximum Sensitivity Substrate (Thermo). The density of the blotting were quantified and analyzed by Image J (NIH).

### Active Rac1 assay

Active Rac1 pull down and detection kit (Thermo) was used to measure active Rac1 in B16 melanoma cells. Briefly, total cell lysates were collected and then mixed with GST-Pak1-PBD and agarose beads. After incubated at 4 °C for 1 hour, the reaction mixture was centrifuged to collect the beads and added reducing buffer to extract protein. Rac1 mAb was used to measure active Rac1. Rac1 in total lysates was detected by western blot. The positive and negative controls were made by adding exogenous GTPrS and GDP to B16 cell lysates.

### Gene expression analysis

Total RNA was extracted from B16 melanoma cells using TRIzol reagent (Invitrogen) according to the manufacturer’s protocol. Reversed transcriptional PCR was performed using random hexamer primers and the TaqMan reverse transcription kit (Applied Bio-systems). Samples were subjected to real-time PCR analysis on an ABI Stepone Sequence Detection System under standard conditions. The primers for mouse E-cadherin and Vimentin were designed using Primer Express software (Applied Biosystems) based on GeneBank accession Number. E-cadherin forward primer 5′-CAGTTCCGAGGTCTACACCTT-3′, reverse primer 5′ –TGAATCGGGAGTCTTCCGAAAA-3′, and Vimentin forward primer 5′-TCCACACGCACCTACAGTCT-3′, reverse primer 5′-CCGAGGACCGGGTC ACATA-3′. Relative mRNA abundance was normalized against GAPDH as the endogenous control (Applied Biosystems).

### Transmission electron microscopy

The cells were dissociated and washed with PBS for three times and then soaked in 2% glutaraldehyde for 24 h. Samples were fixed for 2 h in 1% osmium tetroxide and dehydrated in graded ethanol and embedded in araldite. Sections were observed by a transmission electron microscope (H-765, Hitachi, Tokyo, Japan).

### Statistic

The statistical analyses were performed using SPSS program. All the values were presented as the mean ± SEM, and were analyzed by using t-test. Statistical significance was defined as *p < 0.05, **p < 0.01, ***p < 0.001.

## Additional Information

**How to cite this article**: Tian, L. *et al.* IRGM1 enhances B16 melanoma cell metastasis through PI3K-Rac1 mediated epithelial mesenchymal transition. *Sci. Rep.* **5**, 12357; doi: 10.1038/srep12357 (2015).
